# Communicate hope to motivate the public during the COVID-19 pandemic

**DOI:** 10.1038/s41598-022-06316-2

**Published:** 2022-02-15

**Authors:** Michael Bang Petersen, Lasse Engbo Christiansen, Alexander Bor, Marie Fly Lindholt, Frederik Jørgensen, Rebecca Adler-Nissen, Andreas Roepstorff, Sune Lehmann

**Affiliations:** 1 Department of Political Science, Aarhus University, Aarhus, Denmark; 2 DTU Compute, Technical University of Denmark, Kgs Lyngby, Denmark; 3 Department of Political Science, University of Copenhagen, Copenhagen, Denmark; 4 Interacting Minds Centre, Aarhus University, Aarhus, Denmark; 5 Copenhagen Center for Social Data Science, University of Copenhagen, Copenhagen, Denmark

**Keywords:** Human behaviour, Public health, Epidemiology

## Abstract

How should health authorities communicate to motivate the public to comply with health advice during a prolonged health crisis such as a pandemic? During the SARS-CoV-2 pandemic, for example, people have had to comply with successive restrictions as the world faced multiple races between controlling new waves of the virus and the development and implementation of vaccines. Here, we examine how health authorities and governments most effectively motivate the public by focusing on a specific race: between the Alpha variant and the implementation of the first generation of COVID-19 vaccinations in the winter of 2021. Following prior research on crisis communication, we focus on appeals to fear and hope using communicative aids in the form of visualizations based on epidemiological modelling. Using a population-based experiment conducted in United States ($N = 3,022$), we demonstrate that a hope-oriented visual communication aid, depicting the competing effects on the epidemic curve of (1) a more infectious variant and (2) vaccinations, motivates public action more effectively than a fear-oriented visual communication, focusing exclusively on the threat of the new variant. The importance of the implementation of such hope-oriented messages is further highlighted by cross-national representative surveys from eight countries ($N = 3,995$), which demonstrate that feelings of fear towards the Alpha variant alone were insufficient to activate strong compliance. Overall, these findings provide general insights into the importance of hope as a health communication strategy during the COVID-19 pandemic and beyond.

## Introduction

An epidemic places a heavy toll on the public. This toll is not only related to the impact of the disease but also to the social, mental and financial impact of the non-pharmaceutical interventions used to control the disease^2,5^. Because non-pharmaceutical interventions are costly, it is key for health authorities to engage in communication that increases public understanding and acceptance of the interventions. This is important for securing the interventions’ legitimacy, which is normatively critical in democratic societies, and a prerequisite for the interventions’ effectiveness, as many non-pharmaceutical interventions rely, in part, on people’s voluntary compliance^20^.

In a prolonged crisis such as an epidemic, the costs of interventions accumulate over time, creating feelings of fatigue^10,18^. The communication task for authorities are further challenged by the fact that interventions needs to be re-implemented as new waves of infections build up; for example, related to seasonal changes for viruses with season-dependent transmission or the emergence of new variants of the virus that escape prior immunity.

Focusing on the COVID-19 pandemic, this article asks how health authorities should communicate during an epidemic to generate the necessary understanding and acceptance of interventions.

Theoretically, the literature on crisis communication highlights two emotional targets of communication: Fear and hope. Multiple studies have been arguing that “fear-mongering” has been widespread during the COVID-19 pandemic^4,12^. At the same time, classic work in the psychology of emotions argues that hope is an important precondition for overcoming difficult situations as hope motivates people to take action towards achieving their goals^7,23^. This is consistent with theories of the psychological motivations underlying compliance with protective advice, which emphasize the dual importance of appraisals of threat and appraisals of abilities to cope with the threat^21^. From this perspective, the emotion of hope is an outcome of actionable advice on what to do, how to do it and why to do it^3^. Studies on crisis communication have investigated the relative importance of appeals to hope in relation to, for example, climate-related behavior and find that hope-oriented communication increases support and compliance^6,16,17^. Importantly, these studies also highlight that such appeals should not build hope by downplaying the threat^11^ but by facilitating a sense of efficacy. Studies during the COVID-19 pandemic support the psychological importance of hope-related sentiments. Feelings of self-efficacy, for example, positively predict both compliance with health advice and support for interventions against COVID-19 and does so more strongly than concern about the virus^13,14^. Similarly, studies have shown that other positive feelings such as empathy^20^ and optimism^22^ play a significant role in protective behavior during the pandemic.

On this basis, we test the effectiveness of hope-oriented versus threat-oriented communication during the COVID-19 pandemic. We do so in the context of the pandemic situation in early 2021. At this stage of the pandemic, the arrival of effective vaccines raised global expectations that a return to normal was within reach. As vaccines were rolled out, however, countries across the globe experienced the emergence of a new, more infectious variant of the coronavirus, the Alpha variant. The Alpha variant had a global presence in January 2021 and was expected to be the dominant strain in the countries where it was first discovered within weeks^26^. In terms of both epidemic management and communication, this more infectious variant presented a significant and new challenge.

**Figure 1:**
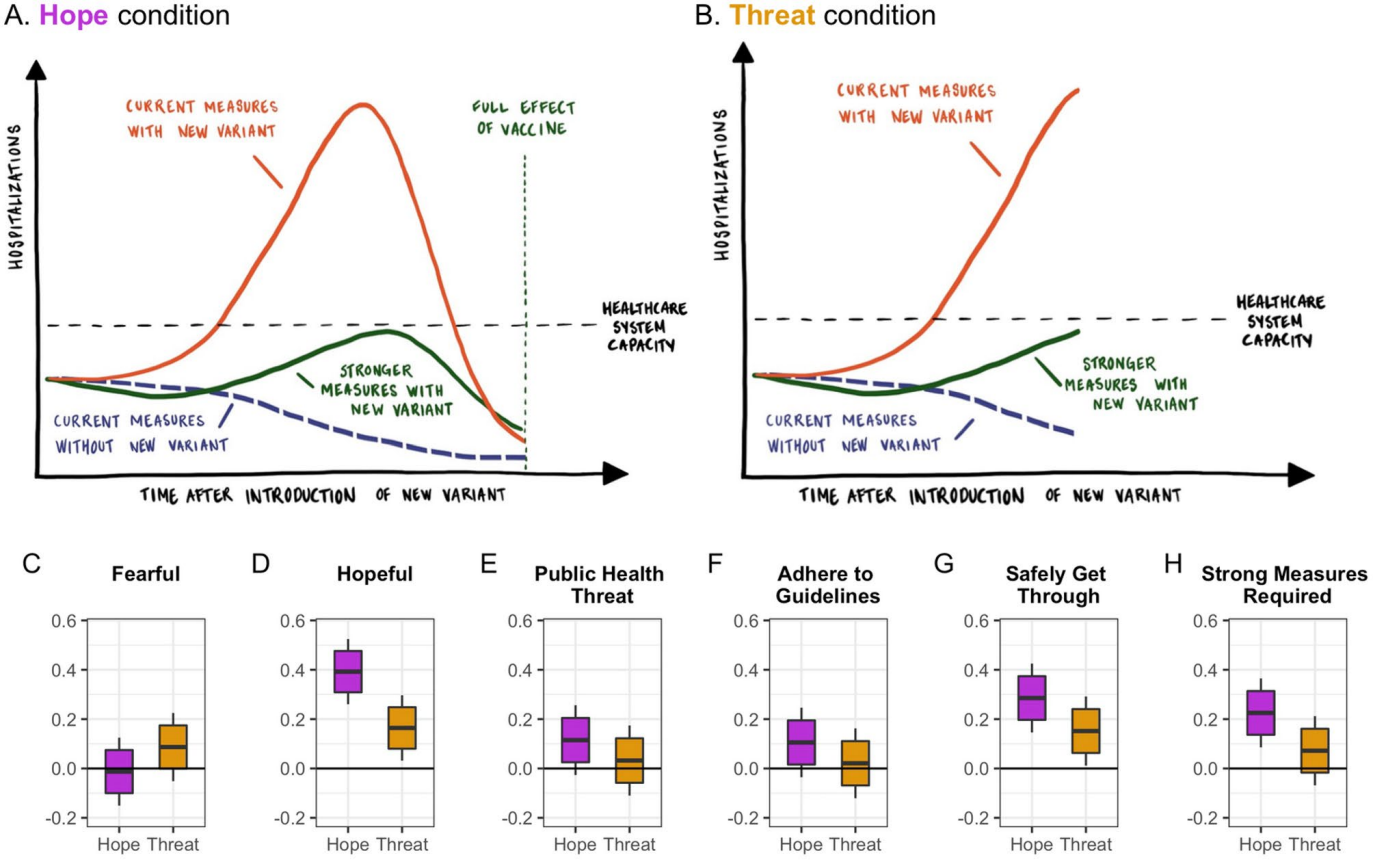
The effectiveness of public health communication with (Panel** A**) and without (Panel** B**) the hope associated with vaccines. Panels (**C**),(**D**) report unstandardized coefficients of marginal effects from OLS regressions that compare responses in the Hope Condition (Panel **A**) and Threat Condition (Panel **B**) to a control condition, respectively. Hinges denote $95\%$ confidence intervals and whiskers denote $99\%$ confidence intervals.

Because of higher infectiousness, the Alpha variant increased the risk of large outbreaks^25^, intensifying the need for citizens to engage in protective behavior^9^, at a time where populations across the globe were strained with pandemic fatigue^10,18^. The challenged posed by the infectious Alpha variant called for intensified communication with the public.

In the first phase of the COVID-19 pandemic, a powerful example of effective hope-oriented communication was a figure illustrating the epidemiological credo of ‘flattening the curve’, which became a global rallying point. This illustration communicated the need for individual and collective action within a conceptual framework of epidemic modelling^1^, in a way that resonated with a large number of people. Yet, in the situation of January 2021 with the Alpha variant, the ‘flatten the curve’ illustration did not adequately communicate either the threat or the hope of coping with the threat.

Epidemiologically, the goal in early 2021 was not to ‘flatten the curve’ in the sense of constantly keeping the number of hospitalized below the capacity of the health care system by moving infections from the present to the future. Instead, the situation involved a race between a variant-driven accelerated rate of infection versus vaccination-driven depletion of the pool of susceptibles. At this stage of the pandemic, the goal of engaging in protective behavior was, therefore, to decrease outbreak size (and associated hospitalizations and deaths) by buying time until vaccines could take effect.

In this critical phase of the pandemic, we tested the effectiveness of a visual communication aid, grounded in epidemiological modelling, in order to increase public support for engaging in stronger protective behavior in the face of the new variant. Consistent with the findings from studies on crisis communication within and outside the pandemic, a core focus of this communication aid was to generate an evidence-based understanding of society’s ability to cope with this emerging threat.

### Testing the Communication Effects of Hope

To create a visual aid for a hope-oriented communication strategy, we built epidemiological models reflecting possible trajectories of the epidemic in one of the first countries where Alpha was identified (Denmark). We modelled three scenarios (Fig 1A): (1) A scenario without Alpha and without the implementation of stronger epidemic control measures. In this scenario the epidemic was effectively controlled until vaccines took full effect (blue dashed curve). (2) A scenario with Alpha but without the implementation of stronger epidemic control measures (the red curve). Here hospitalizations exceeded capacity as Alpha became dominant until the vaccines take full effect. (3) A scenario with Alpha but where stronger interventions were implemented (the green curve). This reduced the infection rate and managed to keep hospitalizations below capacity until the vaccines took full effect. The models were generated from assumptions about infection and vaccination rates and hospital capacity, which at the time was deemed reasonable (see Methods). However, as we return to in the Discussion, several epidemiological factors were not taken into consideration in these scenarios. The function of the scenarios was to provide grounding for the test of hope-oriented communication rather than to provide a full description of the overall pandemic trajectory.

**Figure 2:**
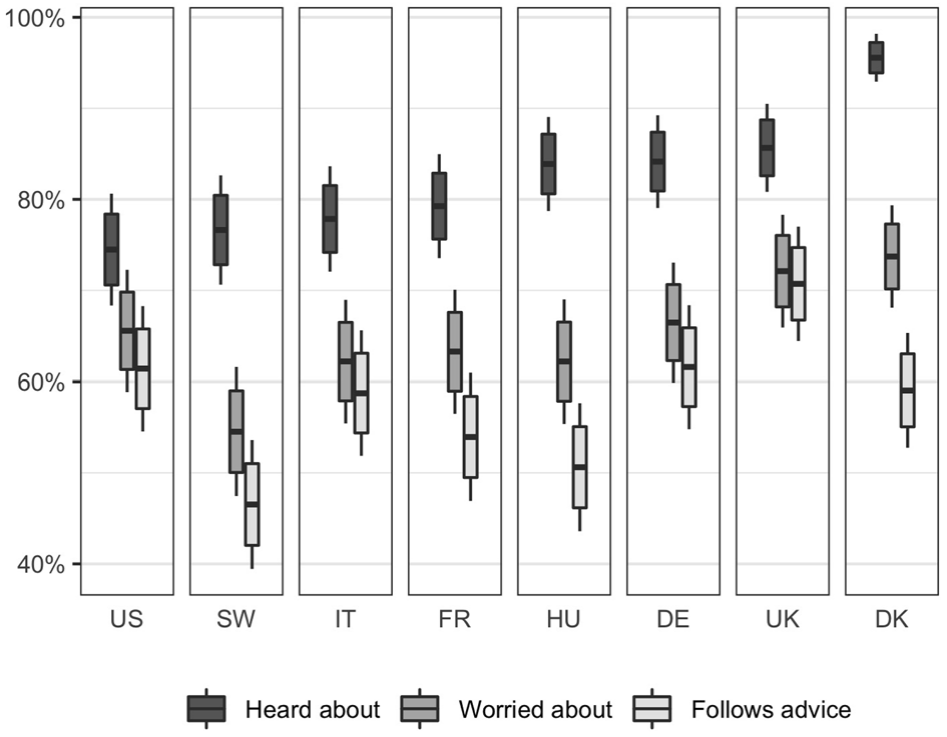
Knowledge, fear and behavior change because of new, more infectious variants. Proportions with associated $95\%$ confidence intervals within each country (Denmark, Sweden, United Kingdom, United States, Italy, France, Germany & Hungary), that agree ‘completely’ or ‘somewhat’ with the statements: (1) ‘I have heard about new variants of the corona-virus that are more infectious’, (2) ‘I am worried about the new, more infectious variants of the corona-virus’, (3) ‘I follow the health authorities’ advice to a greater extent because of new, more infectious variants of the corona-virus’. Total $N = 3,995$.

To assess the communicative effectiveness of this visual aid and associated text, we conducted an online population-based experiment with a sample reflective of the US population in the beginning of February 2021 ($N = 3022$) (for details on all data and models, see code repository at https://github.com/SocialComplexityLab/pandemic_hope). Participants were randomly assigned to one of three conditions: (1) A **control** condition, which simply informed the participants that more infectious variants of the coronavirus were spreading in the US and that authorities may urge citizens to more strongly adhere to the health guidelines. (2) A **hope** condition that displayed the graph from Fig. 1A to the participants and described the displayed epidemic scenarios, emphasizing how the ‘goal is to buy time until vaccines against COVID-19 take effect’. (3) A **threat** condition that displayed and described only the initial half of the graph, focusing exclusively on the threat from the infectious variants without mentioning vaccines and associated hopes (see Fig. 1B). After exposure, respondents were asked to rate whether they found that the information conveyed ‘a fearful message’ and ‘a hopeful message’. Subsequently, respondents completed four 3-item batteries that measured whether the information ‘clearly explained’, helped ‘others understand’ and helped ‘me understand’: (1) ‘why new coronavirus variants are a public health threat’, (2) ‘why [people/they/I] need to adhere more strongly to the health authorities’ guidelines because of new coronavirus variants’, (3) ‘how we as a society can get safely through this pandemic’ and (4) ‘why strong measures are required until vaccines take full effect and drive down infections’. We averaged answers within each battery and *z*-scored the scales using the control group’s mean and standard deviation. For the analyses, we report unstandardized OLS regression coefficients (*b*s) and associated *p*-values.

Figure 1C–H display the marginal effects of the Hope and Threat Conditions relative to the Control Condition. The Threat Condition ($b=.094$, $p=.05$) but not the Hope Condition ($b=-.01$, $p=.78$) is perceived as marginally more fearful, whereas the Hope Condition is perceived as substantially more hopeful ($b=.39$, $p<.001$). Furthermore, the Hope Condition significantly increases the perception of infectious variants as a health threat ($b=.11$, $p<.05$), the motivation to adhere to the guidelines of the health authorities ($b=.11$., $p < .05$), the understanding of how to get safely through the pandemic ($b =.29$, $p< .001$) and why stronger measures are needed ($b=.23$, $p<.001$). The effects of the Threat Condition are for the most part insignificant (beyond effects in Fig. 1D,G) and for Fig. 1D,G,H the marginal effects are significantly stronger for the Hope than the Threat Condition (all *p*s < .05). Furthermore, analyses regression the other outcome measures on observed feelings of hope and fear (controlling for the experimental treatments), demonstrate that feelings of hope ($bs = [.49;.58]$) predict these outcome measures substantatively and significantly stronger than feelings of fear ($bs = [.13;.20]$).

These findings demonstrate that in a phase of the pandemic with significant fatigue, including the hope promised by the advent of vaccines into the communication of future scenarios was effective in motivating stronger adherence to health guidelines and facilitated a better public understanding of the pandemic situation that an exclusive focus on the threat from the new, more infectious variant.

### Is There a need to focus on hope in health communication?

To understand the need for intensified health communication about the Alpha variant, we ran online surveys in eight Western democracies in January 2021. All countries had already identified the variant within their borders at the time: The US, UK, Sweden, France, Italy, Hungary, Germany, France and Denmark. In each country, we surveyed about 500 respondents during January 2021. Respondents were sampled to match the population margins on age, gender, and geographic location for each country and imbalances were addressed via post-stratification. Respondents were asked whether they had heard about the new more infectious variants, whether they were worried about them and whether they as a result adhered more to the authorities’ advice (Fig. 2).

Across all countries, $83 \%$ report that they have heard about new variants. A significantly smaller group ($65 \%$ vs. $83 \%$, $p<.001$, $t(3994)=23.07$), were worried about them and an even smaller fraction ($58 \%$ vs. $65 \%$, $p<.001$, $t(3994)=9.63$) reported to adhere more strongly to health advice as a consequence. These results show that knowledge about new variants did not in itself fuel a sense of threat, which again did not in itself motivate behavior change. These findings highlight the need for re-orienting public health communication towards helping people understand how to cope with threats.

## Discussion

During the COVID-19 pandemic appeals to threat and fear have frequently been used by media, politicians and health authorities in order to motivate the public to engage in protective behavior. Yet, both the literature on crisis communication and psychological studies during the pandemic show that a sense of threat is mainly effective for motivating adequate behavior change, if this sense is coupled with a sense of coping ability or efficacy, resulting in feelings of hope. Providing hope through actionable advice and long-term-oriented communication may be especially important during a prolonged crisis, such as a pandemic, where fatigue may otherwise demotivate publics.

In early 2021, the world experienced a race between the spread of the more infectious Alpha variant of the coronavirus and the implementation of vaccines requiring governments to tightening restrictions in order to buy time. Our results demonstrate that while many had heard about this new variant, fewer had changed their behavior in order limit its spread. To motivate such behavior, we found that communication that not only emphasized the threat of the variant but also society’s ability to cope with the threat generated more support for and understanding of these tighter measures. The present findings thus provides real-world evidence of the importance of hope-oriented messages for public health communication during a critical period of the COVID-19 pandemic.

Three caveats are important to highlight. First, these findings do not suggest that vague reassurances constitute effective communication (i.e., the creation of unfounded hope or what is sometimes colloquially referred to as “hopium”). The stimulus materials in this experiment provided knowledge-based and actionable advice to help navigate an uncertain future. Thus, hope becomes an outcome of empowerment. This is very different from unfounded hope with all the normative problems it implies. Second, even in hope-oriented messaging, it is of importance to be transparent about the involved uncertainties. Research suggests that the transparent declaration of uncertainty or negative information does not decrease trust in the sender^19,24^. In this regard, it is important to note that the materials in the present study was limited in some respects. The modelling was based on the assumption that vaccine-induced herd immunity was reachable against the Alpha variant. Yet, long-term immunity was since challenged by waning immunity from vaccines^8^ and by vaccine-evading variants of the coronavirus^28^. Accordingly, even in countries with very high levels of vaccinations, the experimental stimuli did not adequately capture the long-term uncertainties and complexities in the actual pandemic trajectory. In actual communication from the health authorities, it is important to be transparent about such uncertainties and complexities. Finally, in the experimental study of communication we focused exclusively on average treatment effects (i.e., how the respondents on average reacted). Yet, research as well as actual experiences of many countries during the pandemic has made it clear that societies are heterogeneous and everybody may not react in similar ways. Most importantly, research has revealed significant vaccine hesitancy in many countries^15^ and also found that those who are vaccine hesitant are less likely to react positively to communication from the health authorities^19^. We did not obtain information about vaccine hesitancy among the respondents in the communication experiment and, hence, we cannot assess whether respondents with varying levels of hesitancy reacted differently. Yet, for health authorities, it is important to factor in such heterogeneity when preparing communication.

## Conclusion

The present study investigated health communication in a critical phase of the COVID-19 pandemic that could be characterized as a race between controlling emerging infectious variants and implementing vaccinations. In this situation, motivating the public to engage in protective behavior to buy time was crucial. As demonstrated, a dual communication strategy that emphasized both threat from the new variants and the hope induced by the vaccines was an effective means to this end. The present findings provide public health authorities with a blueprint for health communication in similar future phases of the pandemic (e.g., when implementing new vaccines to deal with waning immunity or vaccine-evading variants). Most generally, the present findings emphasize the importance in public health communication of building hope through actionable advice based on transparent knowledge claims.

## Methods

### Supplementary materials

 All survey materials to reproduce the studies as well as all data and command files to reproduce the presented analyses are available at this open repository: https://github.com/SocialComplexityLab/pandemic_hope.

### Research ethics

 This study complies with Aarhus University’s Code of Conduct as well as the Committee Act of the Danish National Committee of Health Research Ethics, which states that “Surveys using questionnaires and interviews that do not involve human biological material (section 14(2) of the Committee Act)” are exempted from approval (https://en.nvk.dk/how-to-notify/what-to-notify). All participants were above 18 of age and provided written, informed consent to participate.

### Survey sampling

 The experimental study is based on a survey in the United States of America (N=3,022) in February 2021. The data collection was conducted by a survey company, YouGov, on an online panel of adult respondents. Survey respondents were quota-sampled to match the population margins on age, gender, education, geographical location and race. The cross-national observational study is based on surveys in eight countries in January 2021: Denmark (N=586), Sweden (N=475), the United Kingdom (N=502), the United States of America (N=482), Italy (N=492), France (N=482), Germany (N=492)and Hungary (N=484). In each of the eight countries, the survey company, Epinion, sampled adult respondents using online panels. Survey respondents were quota sampled to match the population margins on age, gender, and geographic location for each of the eight countries in our study. We address remaining imbalances by post-stratifying the sample data to match the demographic margins from the population on the same variables that were used for the sampling.

**Figure 3:**
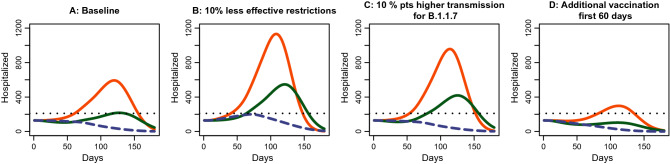
Impact of restrictions, relative transmission and additional vaccination. Each display contains a baseline scenario with B.1.1.7 (red) and without B.1.1.7 (dashed blue) as well as a scenario with B.1.1.7 and $10\%$ more effective restrictions (green). A) reference $R_t=1.0$ and a vaccination rate of 500 persons per day for 60 days and then $5\,000$ per day. B) As A) but with reference $R_t = 1.1$. C) as A) but with relative $R_t$ for B.1.1.7 equal to 1.7 instead of 1.6. D) as A) but with a vaccination rate of 2500 persons per day for the first 60 days. The hospital capacity is 200 beds related to covid-19 (dotted line). All displays are normalized to a population of 1 million.

### Epidemiological modelling

 The model is a SEIR model with nine 10-year age groups up to 80+. It is implemented as a set of 126 ordinary differential equations representing susceptible, two exposed states for each virus variant and two infectious states for each variant, hospitalized, and recovered, dead and vaccinated as terminal states. The graph in the Hope Condition was produced with an effective reproduction number of $R_t=1.0$ for the existing variants, a relative $R_t$ of the Alpha variant of 1.6, the assumption that stronger measures would reduce $R_t$ to 0.9, and a daily vaccine rate of 500 per million population for the first 60 days and then increased to $5\,000$. The models also assumed that $90\%$ of the population wanted to be vaccinated and that vaccines are $95\%$ effective, which at the time seemed a reasonable assumption^27^. The assumption of $90\%$ vaccine acceptance in Denmark was based on available survey data^15^ and is consistent with actual rate of vaccination in Denmark in 2021. As a baseline, the graph used in the communication experiment is reproduced in Fig. 3A. Naturally, however, the specific trajectory of the epidemic depended on a number of factors. Figure 3B–D display the trajectory of the epidemic if the current measures were $10\%$ less effective (Fig. 3B), if the relative Rt for the new variant is 0.1 higher (Fig. 3C) or if the vaccinations were rolled out more quickly in the first 60 days (Fig. 3D). The figures are based on a population of 1 million and a hospital capacity of 200 COVID-19-related hospitalizations (the planned peak capacity in December 2020 in Denmark and coinciding with Alpha becoming a matter of public concern). While the models can account for these varying factors, it is important to note that there were also long-term factors that were not taken into consideration within the six month time window covered by these models. Specifically, the assumption of $95\%$ effectiveness of vaccines were since challenged by emerging issues related to waning immunity^8^ and vaccine-evading variants^28^. Furthermore, within the time window covered by the models, it was assumed that interventions were kept constant, which was not the case. Rather, interventions were gradually lifted as infections went down. Accordingly, epidemiological models such as these should be seen as means to communicate to the public and policy-makers about the epidemic situation within a limited time window.
